# Production of fluorescent and cytotoxic K28 killer toxin variants through high cell density fermentation of recombinant *Pichia pastoris*

**DOI:** 10.1186/s12934-017-0844-0

**Published:** 2017-12-19

**Authors:** Esther Giesselmann, Björn Becker, Manfred J. Schmitt

**Affiliations:** Molecular and Cell Biology, Department of Biosciences and Center of Human and Molecular Biology (ZHMB), Saarland University, 66123 Saarbrücken, Germany

**Keywords:** High cell density fermentation, Fluorescence labelling, Heterologous protein expression, Killer toxin, *Pichia pastoris*, *Saccharomyces cerevisiae*, A/B toxins

## Abstract

**Background:**

Virus infected killer strains of the baker’s yeast *Saccharomyces cerevisiae* secrete protein toxins such as K28, K1, K2 and Klus which are lethal to sensitive yeast strains of the same or related species. K28 is somewhat unique as it represents an α/β heterodimeric protein of the A/B toxin family which, after having bound to the surface of sensitive target cells, is taken up by receptor-mediated endocytosis and transported through the secretory pathway in a retrograde manner. While the current knowledge on yeast killer toxins is largely based on genetic screens for yeast mutants with altered toxin sensitivity, in vivo imaging of cell surface binding and intracellular toxin transport is still largely hampered by a lack of fluorescently labelled and biologically active killer toxin variants.

**Results:**

In this study, we succeeded for the first time in the heterologous K28 preprotoxin expression and production of fluorescent K28 variants in *Pichia pastoris*. Recombinant *P. pastoris* GS115 cells were shown to successfully process and secrete K28 variants fused to mCherry or mTFP by high cell density fermentation. The fluorescent K28 derivatives were obtained in high yield and possessed in vivo toxicity and specificity against sensitive yeast cells. In cell binding studies the resulting K28 variants caused strong fluorescence signals at the cell periphery due to toxin binding to primary K28 receptors within the yeast cell wall. Thereby, the β-subunit of K28 was confirmed to be the sole component required and sufficient for K28 cell wall binding.

**Conclusion:**

Successful production of fluorescent killer toxin variants of *S. cerevisiae* by high cell density fermentation of recombinant, K28 expressing strains of *P. pastoris* now opens the possibility to study and monitor killer toxin cell surface binding, in particular in toxin resistant yeast mutants in which toxin resistance is caused by defects in toxin binding due to alterations in cell wall structure and composition. This novel approach might be easily transferable to other killer toxins from different yeast species and genera. Furthermore, the fluorescent toxin variants described here might likewise represent a powerful tool in future studies to visualize intracellular A/B toxin trafficking with the help of high resolution single molecule imaging techniques.

## Background

The killer phenotype of virus infected *Saccharomyces cerevisiae* strains is elicited by the secretion of antifungal killer toxins which are able to kill sensitive strains of various yeast and fungal species [1]. Due to an intrinsic mechanism of toxin immunity, killer strains are effectively protected against their own toxin and, thereby, possess a growth advantage towards non-killer strains [2, 3]. The vast majority of killer toxins in *S. cerevisiae* is encoded by cytoplasmic dsRNA viruses [3, 4]. In case of K28, the primary gene product of the K28 encoding dsRNA is a preprotoxin whose intracellular processing and maturation within the secretory pathway is mechanistically similar to prepro-α-factor processing in yeast and pro-hormone conversion in higher eukaryotes [3, 5–8].

Maturation of K28 from its precursor resembles a multi-step process initiated by posttranslational import into the lumen of the endoplasmic reticulum (ER) and subsequent removal of the N-terminal signal peptide by signal peptidase cleavage at the ER membrane. Further proteolytic preprotoxin processing in the late Golgi catalysed by the activities of Kex2p and Kex1p results in the formation and final secretion of a disulphide-bonded α/β heterodimeric protein toxin whose β-subunit carries a carboxyterminal ER retention motif (HDEL) which is essential for host cell intoxication and intracellular toxin transport [5, 8, 9].

Internalization of K28 by sensitive yeast cells is realized in a two step mechanism: while α-1,3-linked cell wall mannoproteins are used as primary K28 binding sites at the outer yeast cell surface, the secondary plasma membrane receptor of K28 has recently been identified as the HDEL-receptor Erd2p [10, 11] which ensures endocytotic toxin uptake and retrograde transport through the secretory pathway [9]. After toxin retro-translocation from the ER into the cytosol, the β-subunit of K28 becomes ubiquitylated and proteasomally degraded while α enters the nucleus and causes final cell death [12–15]. Since the dimeric α/β structure is characteristic for A/B toxin family members including clinically relevant representatives like cholera, anthrax and Shiga toxin, K28 represents an attractive model to study A/B toxin trafficking in yeast [16, 17].

In the last decades, mammalian cells have been intensively used to study the mode of intoxification, uptake and intracellular transport of A/B toxins through live cell imaging techniques. To avoid procedures requiring cell fixation and permeabilization (e.g. immunostaining), fluorescently labelled toxins were used to analyze the dynamics of toxin transport in living cells. In this respect, the toxin subunit of interest is either coupled with a fluorophore or fused to a fluorescent protein to microscopically track toxin uptake and intracellular trafficking in real-time [18–20]. In contrast to A/B toxins that penetrate and kill mammalian cells, the respective knowledge on yeast killer toxins is mostly based on genetic screens for mutants with altered toxin sensitivity [21] since fluorescent killer toxin variants for live cell imaging are still lacking due to the pronounced sensitivity of yeast killer toxins to pH changes or fusions to its cytotoxic subunits. In the present study, we used the methylotrophic yeast *Pichia pastoris* as platform for the expression and production of fluorescent variants of killer toxin K28. Tightly controlled cultivation conditions during *P. pastoris* fermentation in a bioreactor led to the secretion of high yields of fluorescent and toxic K28 variants. Further toxin binding studies on sensitive yeast cells likewise constrained that the β-subunit is exclusively responsible for cell wall binding of the fluorescent K28 variants.

## Results and discussion

### Construction and expression of fluorescent K28 variants in *P. pastoris*

**Fig. 1:**
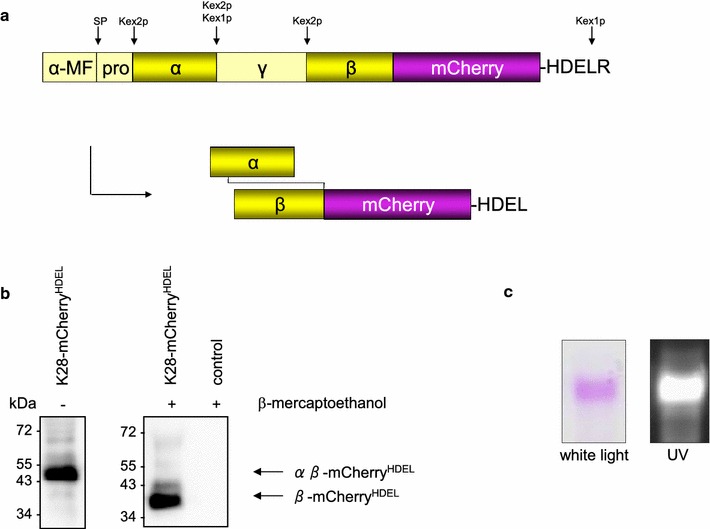
**a** Schematic structure of K28-mCherry^HDEL^. The K28 chimera contains an N-terminal signal peptide derived from the α-mating factor which is removed by signal peptidase (SP) cleavage after ER import. In the *cis*-Golgi, endopeptidase Kex2p cleavage removes the pro- and γ-sequence which leads to the separation of α and β. Both subunits remain connected by a single disulphide which was previously formed in the ER. Carboxypeptidase Kex1p removes the C-terminal arginine residue in a *trans*-Golgi compartment and, thereby, unmasks the HDEL motif. **b** Western blot analysis of secreted K28-mCherry^HDEL^ from *P. pastoris*. Cell-free culture supernatants of the recombinant strain GS115 [pPIC9 K28-mCherry^HDEL^] and the negative control strain GS115 [pPIC9] grown for 120 h in BMM and ×125 concentrated by centrifugal concentrators were separated under non-reducing (left) and reducing (right) conditions by SDS-PAGE and probed with an anti-DsRed antibody. Position and size of the correctly processed toxin is indicated. **c** Detection of fluorescent K28-mCherry^HDEL^. GS115 [pPIC9 K28-mCherry^HDEL^] was grown for 120 h in BMM medium (pH 7) in the presence of protease inhibitors. SDS-PAGE of the ×125 concentrated cell-free culture supernatant under non-reducing conditions shows the pink fluorescent protein and its fluorescence under UV illumination

Fluorescence labelling of proteins and protein toxins is usually achieved by chemically coupling with organic fluorophores or quantum dots [22]. Although the cholera toxin B-subunit was successfully labelled with Cy3 and used for in vivo tracking of its retrograde transport in Vero cells [23], this technique is inappropriate for the viral toxin K28 since the low pH and/or reducing redox milieu during the chemical coupling reaction would immediately inactivate the pH and redox sensitive K28 toxin (Suzuki et al. [32]). Furthermore, since the α-subunit of K28 is highly sensitive to any addition of a tag, we followed another approach and inserted the DNA sequence of the fluorescent protein mCherry between the β-subunit of K28 and its C-terminal HDELR motif. In addition, the native K28 signal sequence was replaced by the α mating factor signal peptide encoded by the expression vector pPIC9 to ensure efficient ER import of the toxin precursor in *P. pastoris* [24, 25]. As illustrated in Fig. 1a, the resulting toxin chimera is predicted to be composed of K28α covalently coupled to β-mCherry^HDEL^ via a single inter-chain disulphide bond.

For in vivo expression of the chimeric K28 variant, the methylotropic yeast *P. pastoris* was used as host to ensure proper posttranslational toxin processing and folding in the eukaryotic secretory pathway, reflecting the natural situation of toxin secretion in K28 killer strains of *S.* *cerevisiae* [26]. Since previous studies already demonstrated that *P. pastoris* is a suitable host for the expression of biologically active wild-type K28 toxin [27], we checked the in vivo processing of the chimeric toxin by analysing the cell-free culture supernatant of recombinant *P. pastoris* GS115 [pPIC9 K28-mCherry^HDEL^] by SDS-PAGE and Western blotting (Fig. 1b). Electrophoretic mobility of K28-mCherry^HDEL^ is in agreement with the calculated size of the K28 chimera. The observed mobility downshift to approximately 11 kDa under reducing conditions is due to a loss of the α-subunit caused by reduction of the disulphide bond which joins α and β-mCherry^HDEL^. Thus, *P. pastoris* was demonstrated to correctly process the K28 variant without being negatively affected by a C-terminal fusion with mCherry. Fluorescence of K28-mCherry^HDEL^ was already visible after SDS-PAGE of a concentrated culture supernatant sample under non-reducing conditions (Fig. 1c). In this study, mCherry was selected because of its insensitivity towards C- and N-terminal fusions and its high acid-stability [28]. In particular, this latter property is of special importance due to the narrow intrinsic pH-optimum of K28 (pH 4.7). Interaction between the secondary toxin receptor Erd2p and the C-terminal HDEL retention motif of K28 only occurs in a mildly acidic pH milieu [11, 29, 30]. The pronounced pH-sensitivity of K28 is probably responsible for the observed lack of killer activity after cultivation of *P. pastoris* in shake flasks for toxin production (data not shown) as no buffer system was capable to stabilize an appropriate pH during longer fermentation periods.

### Fermentation of *P. pastoris* yields in fluorescent and toxic K28 variants

**Fig. 2:**
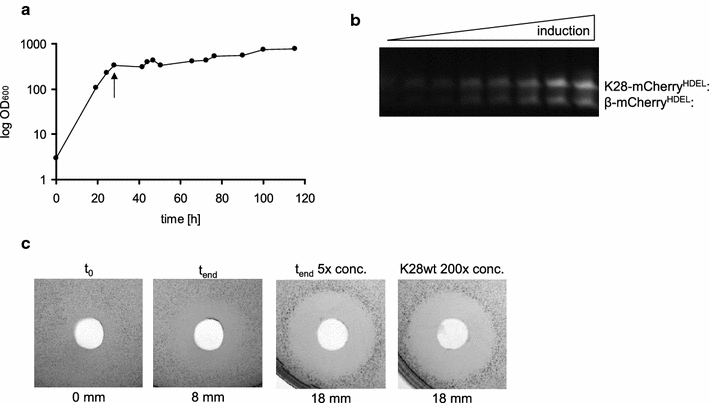
**a** Growth curve of the recombinant *P. pastoris* strain GS115 [pPIC9 K28-mCherry^HDEL^] during fermentation. The starting point of methanol feeding (K28 expression induction) is marked by an arrow. **b** Fluorescence development of mCherry-tagged K28. Cell-free culture supernatant samples from different fermentation time points were separated by SDS-PAGE under non-reducing conditions. Fusion proteins were detected by UV illumination. Position and size of the fusion proteins are indicated. **c** Killer activity of the fermentation product K28-mCherry^HDEL^. Cell-free culture supernatants were tested against the sensitive *S.* *cerevisiae* strain 192.2d in an agar diffusion assay (t_0_ = starting point of methanol feeding; t_end_ = 120 h post methanol induction). Cell-free zones of growth inhibition were determined and killer activity was compared to a ×200 concentrated culture supernatant of the K28 killer strain *S.* *cerevisiae* MS300b

High yields in recombinant protein production after cultivation of *P. pastoris* in shake flasks proved difficult due to limitations in culture volume, oxygen transfer, substrate addition and monitoring [31]. Especially to maintain a constant acidic pH to prevent K28 oligomer formation and inactivation [32], fermentation is the better choice for killer toxin production. Due to the dramatic reduction of K28 killer activity at pH 7.0 and the inadequate fluorescence intensity of mCherry at pH 4.7 (data not shown), medium and culture pH was adjusted to pH 5.3 during the fermentation process. This pH value represents a suitable compromise between efficient in vivo K28 killing activity and sufficient mCherry fluorescence. As soon as the methanol-induced toxin expression started, cultivation temperature was lowered to 20 °C, in turn resembling the temperature optimum for killer toxin production. Usually, a moderate decrease in cultivation temperature can improve heterologous protein production, secretion and folding of fluorescent proteins [33, 34]. Yeast cell growth was controlled by measuring the optical density which achieved values of OD_600_ > 700 (Fig. 2a). To avoid high oxygen concentrations harming K28 production, methanol feedings were reduced as far as possible. As methanol is required to induce protein expression, culture pO_2_ could only be maintained without supplying extra oxygen by regulating the stirring rate during methanol feeding. High oxygen concentrations have been described to induce cell stress and, thereby, to cause the release of proteases, whereas hypoxic conditions positively affect recombinant protein secretion [35–37]. In addition, precisely adjusted methanol feedings during fermentation, which is not possible in shake flask cultures, prevent fluctuations in methanol concentrations [38].

As shown in Fig. 2b, fluorescence of the fermentation product K28-mCherry^HDEL^ was immediately detectable after SDS-PAGE of the cell-free culture supernatant. Again, the heterodimeric K28-mCherry^HDEL^ fusion and the β-mCherry^HDEL^ variant were detectable during fermentation as fluorescent bands under UV illumination, resulting from reductive cleavage of the inter-chain connecting disulphide. In contrast to shaking flask experiments (data not shown), the optimized culture conditions for killer toxin production led to K28 variants with in vivo killing activity (Fig. 2c). In the time course of induction of K28 expression, the culture supernatant showed increasing killer activity against a sensitive yeast tester strain in an agar diffusion assay. The fivefold fermenter concentrate caused the same growth inhibition zone as a 200× concentrated cell-free culture supernatant of a K28 wild-type killer strain of *S.* *cerevisiae* [39], emphasizing the efficiency of bioactive killer toxin production by *P. pastoris* fermentation. Furthermore, this is the first time that a biologically active and fluorescently labelled killer toxin has been expressed and secreted.

Besides mCherry, we also introduced an alternative fluorescent protein that can be easily quenched for future studies. mTFP (monomeric teal fluorescent protein) represents one of the brightest and most photo-stable fluorescent proteins which is likewise less pH sensitive [40]. By using identical fermentation conditions we were able to successfully express a biologically active and fluorescent K28-mTFP^HDEL^ fusion as well as mTFP^HDEL^ in *P.* *pastoris*. To elucidate the influence of the fluorescent proteins and the HDEL-signal on killer activity, additional controls including K28-mCherry^Δ^, mCherry^HDEL^ as well as mCherry^Δ^ were likewise expressed and their in vivo killing ability was tested in toxin activity assays (Table 1). As expected, only wild-type K28 as well as toxin chimera composed of the K28 α/β heterodimer with its C-terminal HDEL-motif showed killer activity. These results also nicely confirm that the α moiety represents the cytotoxic polypeptide of K28 responsible for killing a sensitive target cell [12]. In contrast to α, the C-terminal HDEL-signal is required for toxin interaction with the HDEL-receptor Erd2p at the plasma membrane level, subsequently ensuring endocytotic uptake and retrograde transport of the toxin to the ER [9, 11]. Therefore, K28 lacking a HDEL sequence is neither toxic nor capable to enter sensitive target cells [9]. As summarized in Table 1, the absence of either K28α or the ER targeting motif HDEL prevents efficient killing.

**Table 1:** Killer activity of K28 variants produced by *P. pastoris* fermentation

Toxin variant/control	Toxicity
K28wt	+
K28-mCherry^HDEL^	+
K28-mCherry^Δ^	−
K28-mTFP^HDEL^	+
mCherry^HDEL^	−
mCherry^Δ^	−
mTFP^HDEL^	−

K28 variants which show killing activity in an agar diffusion assay on methylene blue agar plates in form of a zone of growth inhibition (mm) are marked with “+”, whereas the absence of a killing zone is displayed by “−”

### β-subunit of K28 mediates efficient toxin binding to sensitive yeast cells

**Fig. 3:**
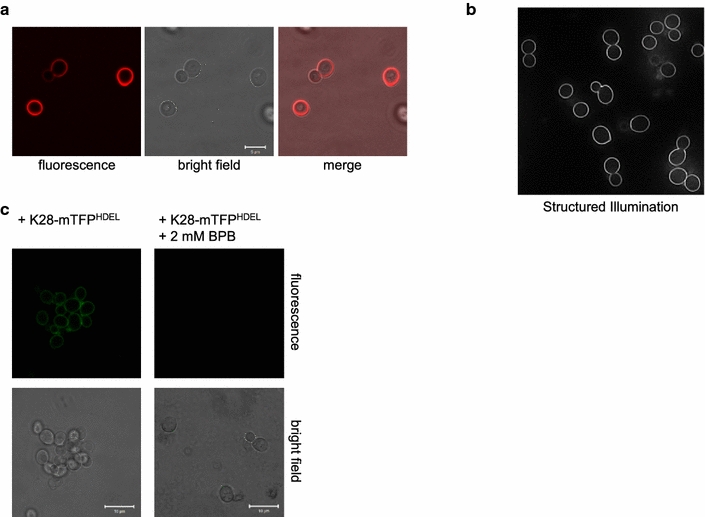
**a** Fluorescence distribution of *S. cerevisiae cells* after incubation with K28-mCherry^HDEL^. *S. cerevisiae* BY4742 was washed twice after 2 h incubation with K28-mCherry^HDEL^ and analyzed by confocal laser scanning microscopy. **b** Same cells analysed by structured illumination microscopy (SIM). The hypersensitive yeast strain 192.2d was treated with K28-mCherry^HDEL^ as described in **a** and subjected to SIM. (**c**) Fluorescence quenching was performed on cells of *S. cerevisiae* BY4742 after incubation with K28-mTFP^HDEL^ as described in **a**, however extracellular mTFP fluorescence was subsequently quenched by the addition of 2 mM bromophenol blue (BPB)

As fluorescently labelled K28 variants possess killer activity (Table 1), the toxin chimera should be taken up by yeast cells and reach the nucleus to finally kill. Consequently, incubation of intact *S.* *cerevisiae* cells with K28-mCherry^HDEL^ led to an intensive fluorescence of the cell periphery (Fig. 3a). Despite extensive variations of the experimental conditions, including changes of the duration, temperature and buffer composition during cell treatment, no intracellular signals were detectable (data not shown). Even by using high-resolution structured illumination microscopy [41] to confirm the fluorescence pattern of K28-mCherry^HDEL^ treated cells of the hypersensitive strain 192.2d [21, 42], only showed strong fluorescence signals around the outer cell periphery (Fig. 3b). To remove this intensive cell surface signal, we tried to quench the extracellular K28-mTFP^HDEL^ fluorescence by using the plasma membrane impermeable quencher bromophenol blue (BPB). BPB is known to efficiently quench the emission of EGFP-fusions. The emission maxima of EGFP and mTFP rarely diverge so that mTFP fluorescence can likewise be quenched by BPB. Consequently, the intracellular killer toxin fraction should be visualized under these conditions. However, as illustrated in Fig. 3a, c, the fluorescence pattern of K28-mTFP^HDEL^ or K28-mCherry^HDEL^ treated cells was not significantly different. BPB addition to cells pre-incubated with K28-mTFP^HDEL^ caused a complete quenching of mTFP fluorescence, indicating that the strong fluorescence observed at the cell periphery is due to K28 binding to the cell wall and not derived from endocytosed intracellular K28 molecules.

The lack of intracellular toxin signals after incubation of sensitive yeast cells with fluorescent K28 variants can somehow be explained by the extremely low amount of toxin molecules that are internalized in vivo. Visualization of compartmental A/B toxin trafficking is even complicated by the low toxin quantity in certain compartments such that toxin visualization by microscopy is often misleading [18]. Based on the high biological activity of A/B toxins, only a few molecules of protein toxins such as ricin or diphtheria toxin are required to kill a cell [43, 44]. A crude estimation for the in vivo toxicity of yeast killer toxin K1 is based on experiments with radioactive labelled K1 in which it was determined that approximately 3 × 10^4^ molecules are required to kill a single cell [45]. However, no information exists about the number of K28 molecules that is needed to reach the nucleus to mediate cell death.

Interestingly, even treatment with the metabolic inhibitor azide, which is known to prevent in vivo cell killing by blocking the energy-dependent K28 internalization step (unpublished data and [39]), did not alter the fluorescence pattern of K28-mCherry^HDEL^ treated cells (data not shown). It, therefore, can be concluded that the observed fluorescence is caused by an energy-independent cell wall binding process of K28. This assumption is further fortified by comparison of different fluorescent K28 derivatives (Table 2). A non-toxic fluorescent K28 variant without a C-terminal HDEL-motif (K28-mCherry^Δ^) caused the same fluorescence pattern as K28-mCherry^HDEL^. As the HDEL-peptide is described to be only crucial for Erd2p-mediated endocytosis and retrograde toxin trafficking [3, 5, 9, 11], the observed K28-mCherry^Δ^ fluorescence in the cell periphery simply arises from energy-independent binding to the outer yeast cell wall.

**Table 2:** Cell wall binding properties of various fluorescent K28 variants

K28 derivates and control proteins	Cell binding/periphery fluorescence
α/β-FP^HDEL^	+
α/β-FP^Δ^	+
β-FP^HDEL^	+
FP^HDEL^	−
FP	−

*FP* fluorescent protein, mCherry or mTFP

To exclude that fluorescent proteins somehow alter the cell wall binding behaviour of K28, yeast cells were analyzed after treatment with fluorescent proteins either containing or lacking the HDEL-motif. Again, the HDEL-motif had no effect on cell binding. Furthermore, fluorescent proteins without any K28 moiety were not able to evoke fluorescence signals at the yeast cell periphery (Table 2). To further substantiate that the β-subunit of K28 is responsible for cell binding, yeast cells were incubated with the β-subunit of K28 fused to mCherry^HDEL^. Incubation with β-mCherry^HDEL^ indeed caused a strong peripheric fluorescence comparable to the fluorescence pattern of K28α/β variants, confirming the β subunit as sole cell binding component of K28.

## Conclusions

Taken together, our data demonstrate that the viral killer toxin K28 can be fluorescently labelled by fusion with fluorescent proteins such as mCherry or mTFP and heterologously expressed in the methylotrophic yeast *P. pastoris* resulting in high-level production and secretion of fluorescent and bioactive toxin chimera which show strong killing activity against sensitive yeast cells. This strategy might be transferable to other yeast killer toxins and used as tool for live cell imaging and high resolution microscopy to analyse cell surface binding. Although no intracellular K28 signals were visible in this study, it might be possible to visualize intracellular toxin trafficking in the future through high resolution single molecule fluorescence microscopy.

## Methods

### Yeast and *E. coli* strains, culture media and conditions

*Pichia pastoris* GS115 (*his4*, ThermoScientific, Waltham, Massachusetts, USA) was selected for heterologous protein expression. For selection of positive transformed clones, cells were cultured in glucose containing histidine d/o medium [27]. For protein expression, cells were initially grown in buffered minimal glycerol medium for biomass production (BMG, 100 mM potassium phosphate (pH 6.0), 0.34% YNB, 1% (NH_4_)_2_SO_4_, 0.00004% biotin, 1% glycerol), followed by minimal methanol medium (BMM, 100 mM potassium phosphate (pH 6.0 or pH 7), 0.34% YNB, 1% (NH_4_)_2_SO_4_, 0.00004% biotin, 0.5% methanol) for induction of gene expression. Cultures were grown at 30 °C for biomass production or at 20 °C for recombinant protein production. The fermentation medium was described previously [35]. *Saccharomyces cerevisiae* 192.2 (*MATα ura3 leu2*) [42] and BY4742 (*MATα his3Δ1 leu2Δ0 lys2Δ0 ura3Δ0*) were grown at 30 °C in complex yeast extract-peptone-dextrose medium. *Escherichia coli* strain TOP 10F′ (*lac*Iq Tn*10* (TetR) *mcr*A Δ(*mrr*-*hsd*RMS*mcr*BC) Φ80*lac*ZΔM15 Δ*lac*X74 *rec*A1*ara*D139 Δ(*ara*-*leu*)7697 *gal*U *gal*K *rps*L *end*A1 *nupG)* was used for all cloning purposes. Bacterial strains were grown in LB media complemented with 100 mg/ml ampicillin.

### Vector construction and transformation

DNA sequences encoding mTFP^HDEL^, mCherry^HDEL^ and mCherry without HDEL motif (mCherry^∆^) were amplified by PCR, for K28-mTFP^HDEL^, K28-mCherry^HDEL^ and K28-mCherry^∆^ by SOE-PCR (Table 3) [46]. Correct sequenced constructs were cloned into pPIC9 (ThermoScientific, Waltham, Massachusetts, USA) via *Xho*I/*Not*I restriction. For *P. pastoris* transformation, plasmids were linearized with *Sal*I to allow integration into the chromosomal *HIS4* locus. *P.* *pastoris* GS115 transformation was performed as described previously [27]. Genomic integration was subsequently confirmed by PCR with appropriate primers.

**Table 3:** PCR primers used in this study

Primer	Sequence (5′–3′)
3′ mCherry	agatctgtcgacgcggccgcTTACTTGTACAGCTCGTCCATGCCG
3′ mCherry^HDELR^	agatctgtcgacgcggccgcTTAGCGTAGCTCATCGTGCTTGTACAGCTCGTCCATGCCGC
3′ mTFP^HDELR^	agatctgtcgacgcggccgcTTAGCGTAGCTCATCGTGCTTGTACAGCTCGTCCATGCCGTC
3′ SOE β-mCherry	CCTCCTCGCCCTTGCTCACCATGCACCTTGCCTCGTCGTCACC
3′ SOE β-mTFP	GTCTCCTCGCCCTTCGTCACCATGCACCTTGCCTCGTCGTCACC
5′ K28 wo SP	agatctctcgagAAAAGAATGCCGACATCTGAGAGACAGCAGGG
5′ mCherry	agatctctcgcgAAAAGAATGCCGACATCTGAGAGACAGCAGGG
5′ mTFP	agatctctcgagAAAAGAATGGTGAGCAAGGGCGAGGAGAC
5′ SOE β-mCherry	GGTGACGACGAGGCAAGGTGCATGGTGAGCAAGGGCGAGGAGG
5′ SOE β-mTFP	GGTGACGACGAGGCAAGGTGCATGGTGAGCAAGGGCGAGGAGAC

Restriction endonuclease cleavage sites used for cloning are shown in small form letters

### Expression, fermentation and purification of K28 variants

Recombinant protein production in *P. pastoris* cultivated in shaking flasks was performed as described previously [27]. After 120 h induction, the culture supernatant was concentrated through 10 kDa cut-off spin columns (Sartorius, Vivaspin 20, Göttingen, Germany).

Fermentation of *P. pastoris* was conducted as described [35] in a Labfors3 bioreactor (Infors, Bottmingen, Switzerland) combined with a MultiTemp III cryostat (Pharmacia, Uppsala, Sweden). Pre-cultures (150 ml fermentation medium) were grown at 30 °C to an optical density (OD_600_) of 10 to 12 and used to inoculate the bioreactor. At the beginning of the fermentation, the bioreactor contained a total volume of 900 ml fermentation medium already completed with hexametaphosphate, trace elements and 0.5 ml anti foam (Breox). 25% ammonia and 20% phosphoric acid were used to maintain the pH at 5.3. The ventilation was adjusted to 2 vvm and the pO_2_ to 25%, regulated by a two stage sequential cascade control (1. stirrer, maximum 1250 rpm; 2. addition of O_2_). After complete glycerol consumption (~ 24 h), indicated by increasing pO_2_, 50% glycerol with trace elements was added (9 ml/h) to increase biomass. After additional 24 h, an optical density at 600 nm of 350–400 was achieved and glycerol feeding terminated. The temperature was decreased to 20 °C for induction because of the temperature sensitivity of K28. Addition of methanol solution with trace elements (1.7 ml/h) led to induction of protein expression. For methanol adaptation, feeding was stopped several times until a limiting growth could be recognized (manual disruption of methanol addition causes a pO_2_ increase). Fermentation process was finished after 100–120 h and culture supernatant was gained by centrifugation (20 min, 10,000 rpm, 4 °C) and stored on ice. Amicon Stirred Cell 8400 (Millipore, Billerica, Massachusetts, USA) was used to concentrate the supernatant (4 °C, 2.5 bar N_2_). Gel filtration chromatography (10% McIlvaine buffer pH 4.8 or 5.3; column material Sephadex G-25 fine (Pharmacia, Uppsala, Sweden)) was performed to desalt the concentrate, followed by sterile filtration allowing long-term storage of K28 variants at − 20 °C or lyophilised and stored at 4 °C in the dark.

### Toxin activity assays

Killer activity of toxin containing culture supernatants was determined in a standard agar diffusion assay on methylene blue agar (MBA) plates (pH 4.7) as previously described [12]. In brief, 1 × 10^6^ sensitive cells of *S.* *cerevisiae* 192.2d were embedded into 15 ml MBA. 100 µl of culture supernatant or desalted toxin concentrate was pipetted into wells (9 mm in diameter) which were cut into the agar. For comparison, culture supernatant of *S.* *cerevisiae* MS300b [47] was concentrated 200× and filled into the well. After 4 days at 20 °C, the diameter of the resulting cell-free zone of growth inhibition surrounding the well was measured, which is proportional to the logarithm of killer toxin activity.

### Fluorescence microscopy

Exponentially growing *S.* *cerevisiae* cells were harvested, washed with incubation buffer (10% McIlvaine buffer pH 4.7, 10 mM glucose, 10 mM CaCl_2_) and resuspended in 250 µl incubation buffer containing fluorescent toxin variants (1.4 µg/ml). After 2 h incubation (20 °C, 60 rpm) and three washing steps with incubation buffer, cells were immobilized on concanavalin A coated slides. Zeiss LSM 510 META (mCherry: 543 nm excitation, HFT 514, NFT 545, LP 560 filter, mTFP: 488 nm excitation, HFT 488, NFT 490, BP 500–530 filter) was used for obtaining confocal images. For image acquisition and processing, Zeiss LSM Image Examiner software was employed. The setup for Structured Illumination Microscopy was a prototype from Zeiss. Images were taken with a 63× Plan-Apochromat (NA 1.4) with excitation light of 561 nm wavelength and then processed for SIM to obtain higher resolutions. Zen2009 software was used for acquisition and processing of the images for higher resolution (Zeiss, Oberkochen, Germany).

### Western analysis

SDS-PAGE was performed under non-reducing conditions in 10% Tris-Tricine gels unless otherwise indicated. Semi-dry blotting onto PVDF membranes was carried out in transfer buffer (25 mM Tris, 190 mM glycin, 0.1% SDS, 20% methanol). To allow mCherry detection by immunoblotting and UV illumination (ChemiDoc XRS system, Biorad, Hercules, California, USA) samples were not heated before application. For toxin detection, blots were incubated with primary anti-DsRed antibodies (Clontech, Kyoto, Japan) and secondary HRP-conjugated goat anti-rabbit antibodies (Sigma, St. Louis, Missouri, USA). Chemiluminescent detection was obtained by the addition of “Western Lightning Plus-ECL” (PerkinElmer, Waltham, Massachusetts, USA).

## Abbreviations

ER: endoplasmic reticulum
SP: signal peptidase
BPB: bromophenol blue
SIM: structured illumination microscopy
Vvm: vessel volumes per minute
